# Physiological and genomic features of *Paraoceanicella profunda* gen. nov., sp. nov., a novel piezophile isolated from deep seawater of the Mariana Trench

**DOI:** 10.1002/mbo3.966

**Published:** 2019-11-19

**Authors:** Ping Liu, Wanzhen Ding, Qiliang Lai, Rulong Liu, Yuli Wei, Li Wang, Zhe Xie, Junwei Cao, Jiasong Fang

**Affiliations:** ^1^ Shanghai Engineering Research Center of Hadal Science and Technology College of Marine Sciences Shanghai Ocean University Shanghai China; ^2^ National Engineering Research Center for Oceanic Fisheries Shanghai Ocean University Shanghai China; ^3^ Key Laboratory of Marine Genetic Resources Ministry of Natural Resources of PR China State Key Laboratory Breeding Base of Marine Genetic Resources Fujian Key Laboratory of Marine Genetic Resources Third Institute of Oceanography Xiamen China; ^4^ Laboratory for Marine Biology and Biotechnology Qingdao National Laboratory for Marine Science and Technology Qingdao China; ^5^ Department of Natural Sciences Hawaii Pacific University Honolulu HI USA

**Keywords:** genome sequencing, high‐pressure adaptation, *Paraoceanicella profunda*, piezophilic, polyphasic taxonomy

## Abstract

A novel piezophilic alphaproteobacterium, strain D4M1^T^, was isolated from deep seawater of the Mariana Trench. 16S rRNA gene analysis showed that strain D4M1^T^ was most closely related to *Oceanicella actignis* PRQ‐67^T^ (94.2%), *Oceanibium sediminis* O448^T^ (94.2%), and *Thioclava electrotropha* ElOx9^T^ (94.1%). Phylogenetic analyses based on both 16S rRNA gene and genome sequences showed that strain D4M1^T^ formed an independent monophyletic branch paralleled with the genus *Oceanicella* in the family *Rhodobacteraceae*. Cells were Gram‐stain‐negative, aerobic short rods, and grew optimally at 37°C, pH 6.5, and 3.0% (*w*/*v*) NaCl. Strain D4M1^T^ was piezophilic with the optimum pressure of 10 MPa. The principal fatty acids were C_18:1_ *ω*7*c*/C_18:1_ *ω*6*c* and C_16:0_, major respiratory quinone was ubiquinone‐10, and predominant polar lipids were phosphatidylglycerol, phosphatidylethanolamine, and an unidentified aminophospholipid. The complete genome contained 5,468,583‐bp with a G + C content of 70.2 mol% and contained 4,855 protein‐coding genes and 78 RNA genes. Genomic analysis revealed abundant clues on bacterial high‐pressure adaptation and piezophilic lifestyle. The combined evidence shows that strain D4M1^T^ represents a novel species of a novel genus in the family *Rhodobacteraceae*, for which the name *Paraoceanicella profunda* gen. nov., sp. nov. is proposed (type strain D4M1^T^ = MCCC 1K03820^T^ = KCTC 72285^T^).

## Graphical abstract

A novel piezophilic alphaproteobacterium, strain D4M1^T^, was isolated from deep seawater of the Mariana Trench. The combined evidence shows that it represents a novel species of a novel genus in the family *Rhodobacteraceae*, for which the name *Paraoceanicella profunda* gen. nov., sp. nov. is proposed. The complete genome contained 5,468,583‐bp with a G + C content of 70.2 mol% and contained 4,855 protein‐coding genes and 78 RNA genes. Genomic analysis revealed abundant clues on bacterial high‐pressure adaptation and piezophilic lifestyle.

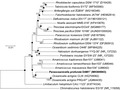

## 1 INTRODUCTION

The deep sea, accounting for approximately 75% of the total ocean volume and hosting 62% of the global biosphere (Fang, Zhang, & Bazylinski, 2010), is a reservoir of remarkably diverse archaea and bacteria. The extreme physical–chemical factors (high salinity, high pressure, and low temperature) in the deep sea may have considerable influences on microbial life. For example, high pressure, the most unique physical parameter in the deep sea, decreases membrane permeability and stability, impedes energy metabolism, and inactivates proteins (Jebbar, Franzetti, Girard, & Oger, 2015; Picard & Daniel, 2013). Thus, piezophiles must evolve physiological and genomic adaptations to grow under high‐pressure conditions. Microorganisms use different strategies to thrive in high‐pressure conditions, such as synthesizing piezolytes, improving permeability and stability of cell membrane, regulating gene expression, and modifying genome features (Oger & Jebbar, 2010; Simonato et al., 2006). Despite the fact that greater than 88% of the ocean's biosphere is above 10 MPa (water depths of 1,000 m or more), a limited number of piezophiles have been isolated and characterized (Picard & Daniel, 2013; Zhang, Wu, & Zhang, 2018).

During our recent campaign of investigating the diversity of culturable microbes in the deep ocean, we isolated a novel piezophilic bacterium D4M1^T^, which was closely related to the species in the family *Rhodobacteraceae* within the class *Alphaproteobacteria*. The family *Rhodobacteraceae* (type genus, *Rhodobacter*) contains more than 130 genera (http://www.bacterio.net/), many members of which have been isolated from the marine environment (Albuquerque, Rainey, Nobre, & da Costa, 2012; Chang et al., 2018; Chang, Meng, Du, & Du, 2019). Additionally, some members have been isolated from deep‐sea environment, such as members belonging to the genera *Acidimangrovimonas* (Jiang, Xu, Shao, & Long, 2014), *Brevirhabdus* (Wu et al., 2015), *Celeribacter* (Lai, Cao, Yuan, Li, & Shao, 2014), *Citreicella* (Lai et al., 2011), *Marinibacterium* (Li, Lai, et al., 2015), *Meridianimarinicoccus* (Ren et al., 2019), *Pararhodobacter* (Lai, Liu, Yuan, Xie, & Shao, 2019), *Profundibacterium* (Lai et al., 2013), and *Thiobacimonas* (Li, Tang, Liu, & Jiao, 2015). In this study, the marine bacterial strain D4M1^T^ was characterized using a polyphasic approach, along with the genome sequence analysis and high‐pressure adaptation.

## 2 MATERIALS AND METHODS

### 2.1 Strains and culture conditions

A deep seawater sample was collected at a depth of 10,890 m from the Mariana Trench (142.4°E, 11.4°N; site MT) in December 2016. The sample (1 ml) was serially diluted with 10 ml sterilized natural seawater and spread onto a selective D4 agar medium (1.0 L seawater, 0.2 g yeast extract, 3.0 g HEPES, 2.0 g xylose, 17.0 g agar, and pH 7.0) under atmospheric pressure. Subsequently, a white‐colored strain D4M1^T^ was isolated by restreaking single colonies onto D4 agar plates at 10°C. The strains grew well on marine agar 2216 (MA; BD Difco) or in marine broth 2216 (MB; BD Difco) medium and were routinely cultivated on MA or MB in this study, unless noted otherwise. Stock cultures were stored at −80°C with 20% (*v*/*v*) glycerol. The phylogenetically related type strains, *Oceanicella actignis* DSM 22673^T^ (=PRQ‐67^T^), *Thioclava electrotropha* DSM 103712^T^ (=ElOx9^T^), and *Oceanibium sediminis* MCCC 1H00233^T^ (=O448^T^), were obtained from the Leibniz Institute DSMZ–German Collection of Microorganisms and Cell Cultures (DSMZ) and Marine Culture Collection of China (MCCC), respectively.

### 2.2 DNA extraction, genomic, and phylogenetic analyses

Genomic DNA was extracted from liquid cultures of strain D4M1^T^ after being cultivated in MB for 36 hr using the ChargeSwitch® gDNA Mini Bacteria Kit (Life Technologies) according to the manufacturer's instructions. The 16S rRNA gene of strain D4M1^T^ was amplified and sequenced by using conserved primers Bac8F (5′‐AGAGTTTGATCATGGCTCAG‐3′) and U1492R (5′‐GGTTACCTTGTTACGACTT‐3′), as reported previously (Cao et al., 2016). The 16S rRNA gene sequence was identified using global alignment algorithm implemented at the EzBioCloud server (https://www.ezbiocloud.net/; (Yoon et al., 2017)). Phylogenetic analysis of 16S rRNA gene was conducted with MEGA 5.0 package (Tamura et al., 2011), using the Kimura two‐parameters model with the neighbor‐joining (Saitou & Nei, 1987) and maximum‐likelihood (Felsenstein, 1981) algorithms, respectively. The tree topology was calculated by bootstrap analysis based on 1,000 bootstraps.

Purified genomic DNA was quantified by TBS‐380 fluorometer (Turner BioSystems Inc.). The complete genome was sequenced using a combination of Pacific Biosciences (PacBio) *RS* and Illumina sequencing platforms (Shanghai Majorbio Bio‐pharm Technology Co., Ltd.). For PacBio sequencing, 8–10 k insert whole‐genome shotgun libraries were generated and sequenced on a PacBio *RS* instrument using standard methods. For Illumina sequencing, 500 bp paired‐end library were generated and sequenced using Illumina Hiseq Xten. The genome was assembled using Velvet assembler (v1.2.09) with a kmer length of 17 and “PacBioToCA with Celera Assembler” pipeline (Chin et al., 2013; Koren et al., 2012) with both the PacBio reads and Illumina reads. The genome sequences of *Thioclava electrotropha* Elox9^T^ (NBXF00000000), *Thioclava pacifica* DSM 10166^T^ (AUND00000000), *Rhodobacter megalophilus* DSM 18937^T^ (FZOV00000000), *Rhodobacter johrii* JA192^T^ (PZZW00000000), *Paenirhodobacter enshiensis* DW2‐9^T^ (JFZB00000000), *Oceanicella actignis* CGMCC 1.10808 (FRDL00000000), and *Oceanibium sediminis* O448^T^ (QGNX00000000) were obtained from the NCBI website. The *Oceanicella actignis* DSM 22673^T^ genome sequence (IMG Genome ID: 2593339287) was downloaded from the Genome portal of the Joint Genome Institute (JGI) (http://genome.jgi.doe.gov/). The genomic DNA G + C content was estimated from the genome sequence. A whole‐genome‐based phylogenetic tree was reconstructed based on the whole‐genome protein sequences using CVTree3 (http://tlife.fudan.edu.cn/cvtree/cvtree/) with *K*‐value = 6 (Zuo & Hao, 2015). The genomic analyses were performed as described previously (Cao, Lai, Yuan, & Shao, 2015) using the tools available on the Integrated Microbial Genomes (IMG) server (https://img.jgi.doe.gov) (Chen et al., 2019).

### 2.3 Phenotypic, physiologic, and biochemical analyses

Images of cells of strain D4M1^T^ were obtained with a transmission electron microscopy (JEM‐1230; Jeol) after glutaraldehyde prefixation and uranyl acetate staining of cells grown on MA at 37°C for 30 hr. Growth characteristics were determined by the measurement of optical density at 600 nm (OD_600_) using a NanoDrop 2000c spectrophotometer (Thermo Scientific). The growth temperature was evaluated at 4, 10, 20, 25, 30, 37, 40, 45, and 50°C in duplicates in 10 days. The salinity range (0, 0.5, and 1%–10% (intervals of 1%) of NaCl, *w*/*v*) and pH range (pH 4.0–11.0 (intervals of 1 unit), added with 20 μmol/L HOMOPIPES, MES, PIPES, HEPES and CAPS buffers, respectively) were investigated as previously described in duplicates (Lai et al., 2014). Gram‐staining, oxidase, and catalase activity were carried out according to the test procedures described by Dong and Cai (2001). Growth under anaerobic condition was tested in LB liquid medium (for fermentation) and in LB supplemented with Na_2_SO_4_ or NaNO_3_ (10 mmol/L, for anaerobic respiration) with oxygen‐free N_2_ gas phase (200 kPa) in sealed sterile vials at 37°C for 7 days. Poly‐*β*‐hydroxybutyrate (PHB) production was determined by using Nile blue A staining and an upright fluorescence microscope (ECLIPSE Ni‐U; Nikon) according to a previous study (Ostle & Holt, 1982). Determination of the hydrostatic pressure range for growth was carried out in hydrostatic pressure vessels under a pressure range of 0.1–80 MPa (intervals of 10 MPa) at the optimal temperature (37°C), with oxygen‐saturated Fluorinert (FC‐40, 3M Company. 25% of total volume) added to supply oxygen (Kato, Sato, & Horikoshi, 1995). Other biochemical tests were carried out using API 20NE, API ZYM strips (bioMérieux) and GEN III microplates by Biolog system (Biolog Microstation™) according to the manufacturer's instructions. Some tests in API strips, such as reduction of nitrate, fermentation of D‐glucose, hydrolysis of aesculin, and utilization of citrate, were also re‐examined by conventional biochemical identification as described by Dong and Cai (2001).

### 2.4 Chemotaxonomic analysis

The fatty acid and polar lipid profiles of strain D4M1^T^ were analyzed on exponential growth phase of cultures grown in MB at 37°C for 48 hr. Fatty acids in whole cells were saponified, extracted, and methylated using the standard protocol of Microbial IDentification Inc. (MIDI, Sherlock Microbial Identification System, version 6.0B). The fatty acids were analyzed by gas chromatography (GC, Agilent Technologies 6850) and identified by using the TSBA 6.0 database of the Microbial Identification System (Sasser, 1990). Polar lipids were extracted from 100 mg of freeze‐dried cells using a chloroform/methanol system, separated by two‐dimensional thin‐layer chromatography (TLC) on silica gel 60 F_254_ plates (Merck), and then identified with molybdophosphoric acid as the spray reagent according to a previously described method (Tindall, Sikorski, Smibert, & Krieg, 2007). The fatty acid and polar lipid profiles of reference strains *Oceanicella actignis* DSM 22673^T^ and *Thioclava electrotropha* DSM 103712^T^ were performed in parallel with strain D4M1^T^ under the same condition. The respiratory quinone was extracted from freeze‐dried cells with chloroform/methanol (2:1, *v*/*v*) and evaporated to dryness at 35°C. The extracts were resuspended in chloroform/methanol (2:1, *v*/*v*) and subsequently purified by TLC on GF_254_ silica gel plates (Branch of Qingdao Haiyang Chemical Co. Ltd.) with n‐hexane/ether (17:3, *v*/*v*). The respiratory quinones were measured by HPLC‐MS system (Agilent) (Wu et al., 2015).

## 3 RESULTS AND DISCUSSION

### 3.1 Phylogenetic and phylogenomic analyses

**Figure 1:**
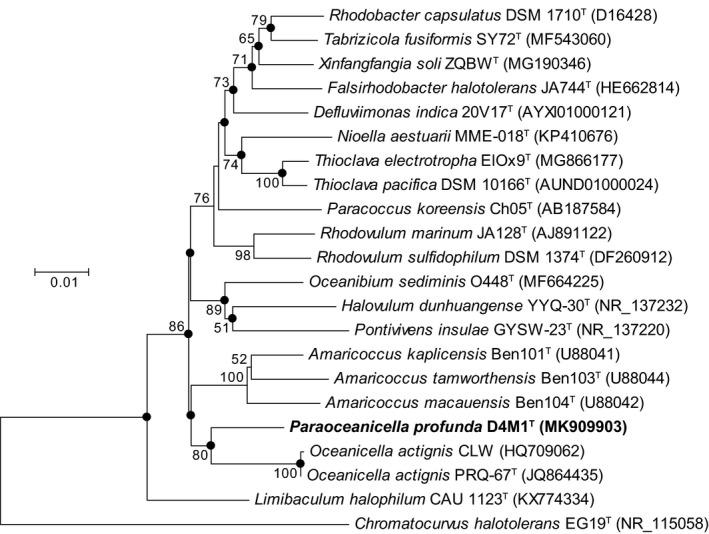
Neighbor‐joining tree showing the phylogenetic positions of strain D4M1^T^ and related species, based on 16S rRNA gene sequence. *Chromatocurvus halotolerans* EG19^T^ was used as outgroup. Filled circles indicate nodes that were also recovered in the maximum‐likelihood (Figure A1) tree for the same sequences. Bootstrap values (expressed as percentages of 1,000 replications) greater than 50% are shown at branch nodes. Bar, 0.01 nucleotide substitution rate (*K*_nuc_) units

**Figure A1:**
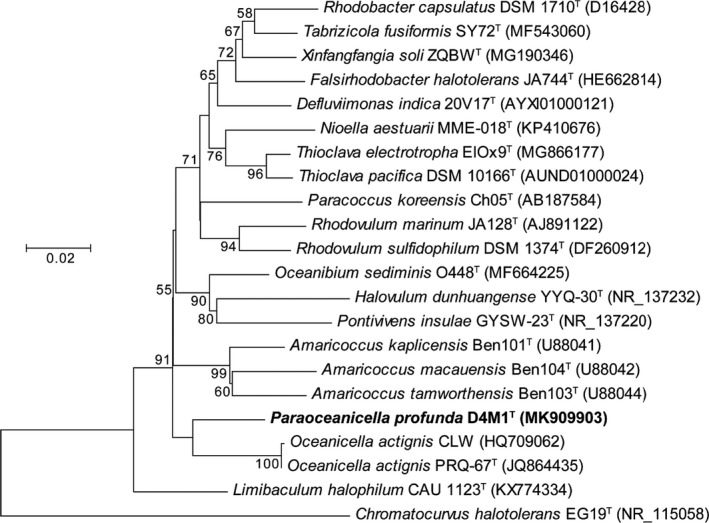
Maximum‐likelihood tree showing the phylogenetic positions of strain D4M1^T^ and related species, based on 16S rRNA gene sequence. *Chromatocurvus halotolerans* EG19^T^ was used as outgroup. Bootstrap values (expressed as percentages of 1,000 replications) are shown at branch nodes. Bar, 0.02 nucleotide substitution rate (*K*_nuc_) units

16S rRNA gene sequence analysis showed that strain D4M1^T^ had the highest 16S rRNA gene sequence similarity of 94.2% with *Oceanicella actignis* PRQ‐67^T^ and *Oceanibium sediminis* O448^T^, followed by *Thioclava electrotropha* ElOx9^T^ (94.1%). Genera are generally described as agglomerates of nodal species and internodal strains (Gillis, Vandamme, De Vos, Swings, & Kersters, 2001), for which similarity values around 94.5%–95% are commonly used for genus differentiation (Ludwig et al., 1998; Yarza et al., 2014). Based on these criteria, strain D4M1^T^ likely represent a novel genus in the family *Rhodobacteraceae*. Phylogenetic analysis based on 16S rRNA gene sequence showed that strain D4M1^T^ formed an independent monophyletic branch paralleled with the genus *Oceanicella* within the family *Rhodobacteraceae*, suggesting that it may represent a novel genus within the family *Rhodobacteraceae* (Figure 1 and Figure A1).

**Figure 2:**
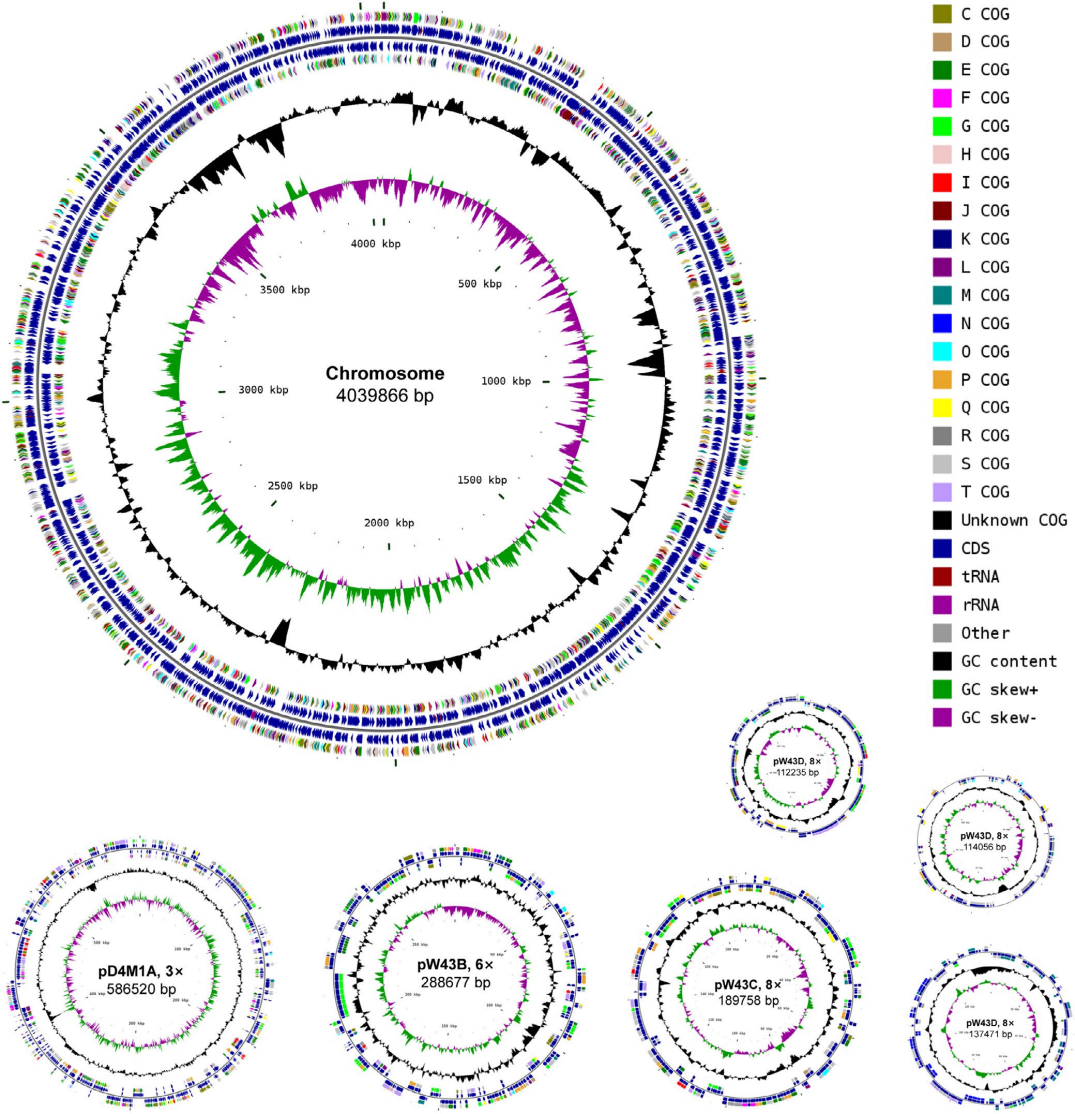
Circular maps of the chromosome and six plasmids of strain D4M1^T^. Plasmids pD4M1A, pD4M1B, pD4M1C, pD4M1D, pD4M1E, and pD4M1F are shown at 3, 6, 8, 8, 8, and 8 × scale, respectively, relative to the chromosome scale. From the outside to the center: protein‐coding genes on forward strand (color by COG categories), total genes on forward strand, total genes on reverse strand, protein‐coding genes on reverse strand, G + C content, and G + C skew

**Figure 3:**
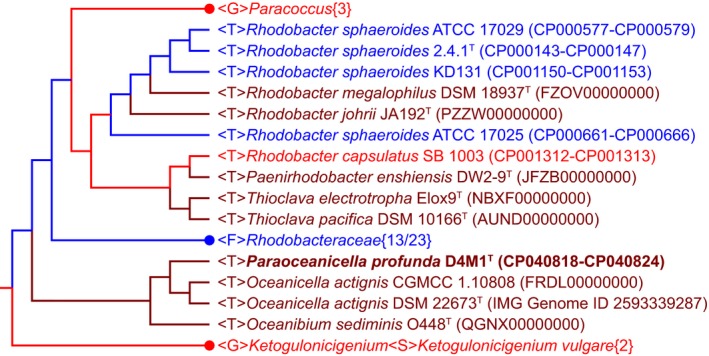
Whole‐genome based phylogenetic tree constructed using CVTree3 showing the phylogenetic relationship of strain D4M1^T^ with reference species in the family *Rhodobacteraceae*. The tree constructed using protein sequences, and *K* = 6. Numbers in bracket stand for the numbers of strains used for phylogenetic analysis. Abbreviation: <F>, Family; <G>, Genus; <S>, Species; <T>, sTrain

Phylogenomic analysis, previously suggested to provide a better taxonomic framework at the genus and higher levels (Chun et al., 2018), was further carried out to provide a better taxonomic characterization. A total of 2.36 Gb of clean data were generated from the genome sequencing of D4M1^T^. The final assembly has 431‐fold coverage for the complete genome, which contains 5,468,583‐bp with a G + C content of 70.2 mol%. The complete genome consists of a circular chromosome of 4,417,125 bp and six plasmids ranging from 112,235 bp to 586,520 bp in length (Table 1 and Figure 2). The assembled and annotated genome of D4M1^T^ has been deposited in GenBank (accession numbers: CP040818–CP040824) and JGI portal (GOLD ID: Gp0432545; IMG Taxon ID: 2828513066). A whole‐genome‐based phylogenomic tree (Figure 3) showed that strain D4M1^T^ formed an independent monophyletic branch within the family *Rhodobacteraceae*. This result supports that strain D4M1^T^ represents a genus‐level taxon in agreement with the result of 16S rRNA gene phylogeny.

**Table 1:** General features of the complete genome sequence of strain D4M1^T^

Content	Chromosome	Plasmids					
		pD4M1A	pD4M1B	pD4M1C	pD4M1D	pD4M1E	pD4M1F
Size (bp)	4039866	586520	288677	189758	137471	114056	112235
G + C content (mol%)	70.0	71.0	71.5	71.7	71.1	61.2	71.4
Protein‐coding genes	3588	525	241	171	111	124	95
Average gene size (bp)	940	967	1037	1001	1028	809	1011
Coding density (%)	83.5%	86.6%	86.6%	90.2%	83.0%	88.0%	85.6%
Gene assigned to COG	2996	462	211	154	74	84	76
tRNA	54	3	0	0	0	0	0
rRNA operon (23S, 16S and 5S)	3	1	0	0	0	0	0
ncRNA	9	0	0	0	0	0	0
GenBank accession	CP040818	CP040819	CP040820	CP040821	CP040822	CP040823	CP040824

### 3.2 Morphology and physiology properties

**Figure A2:**
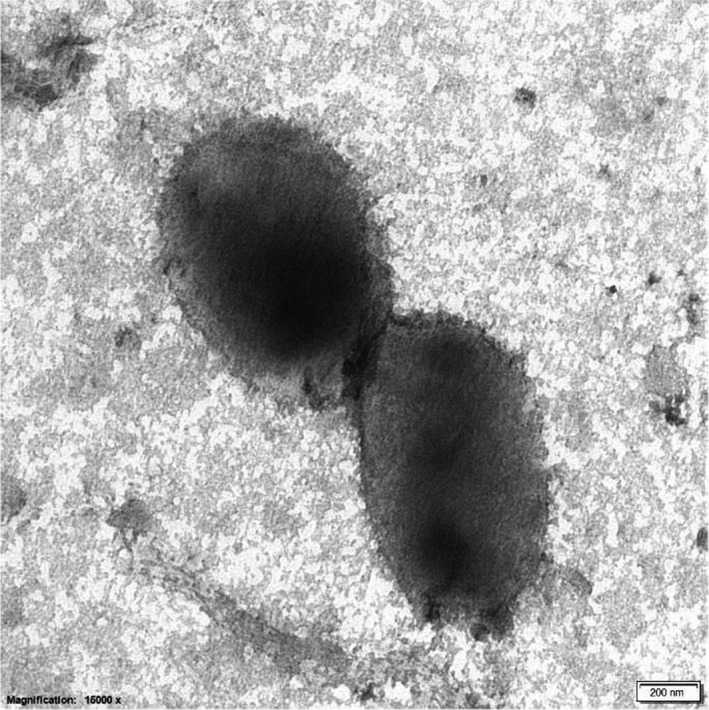
Transmission electron microscopy image of cell of strain D4M1^T^ grown on MA medium at 35°C for 24 hr

**Figure A3:**
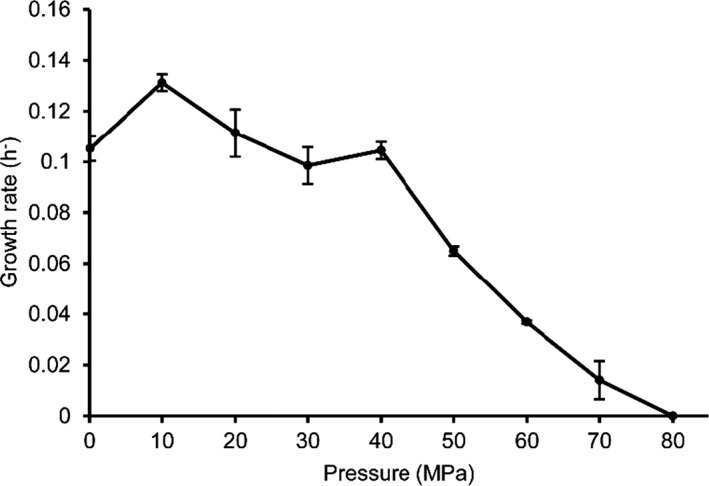
Growth rate of strain D4M1^T^ under different pressures at 35°C

Cells of strain D4M1^T^ are Gram‐stain‐negative, oxidase‐ and catalase‐positive, aerobic, short rods (1.0–1.5 × 0.6–0.8 μm, Figure A2). Growth of the novel strain occurs between pH 5.0–8.0 (optimum 6.5), 10–45°C (optimum 37°C), and in the presence of 0.5%–8.0% (*w*/*v*) NaCl (optimum 3.0%). The novel strain contains poly‐*β*‐hydroxybutyrate (PHB) inside the cells. Strain D4M1^T^ is piezophilic, with the optimum growth pressure of 10 MPa and tolerance up to 70 MPa (Figure A3). Anaerobic growth was not observed in LB medium nor in LB medium supplemented with 10 mmol/L of Na_2_SO_4_ or NaNO_3_. Results of carbon utilization (Biolog GEN III), API ZYM and 20NE tests are given in Table 2 and the species description below. Strain D4M1^T^ is distinguishable from their closest relatives in physiological characteristics as shown in Table 2.

**Table 2:** Differentiating characteristics between strain D4M1^T^ and its close relatives

	1	2	3	4
Growth at 10°C	+	−	+	−
Optimum temperature	37	50	35	37
Growth in 8% NaCl	+	+	+	−
Growth at pH 5	+	−	−	−
G + C content (mol %)	70.2	72.3	63.8	65.8
Enzyme activity				
Lipase (C14)	w	+	−	+
*α*‐Chymotrypsin	+	−	−	−
*α*‐Galactosidase	−	−	+	+
*β*‐Galactosidase	−	−	+	+
*β*‐Glucuronidase	−	−	+	+
*α*‐Glucosidase	w	−	+	+
*β*‐Glucosidase	−	−	+	+
Utilization of:				
D‐Glucose	w	−	−	w
L‐Arabinose	w	−	+	+
D‐Mannose	−	−	w	+
D‐Mannitol	**+**	−	+	+
D‐Maltose	−	−	+	+
Potassium gluconate	+	−	+	+
Malic acid	w	−	+	+
Trisodium citrate	−	−	+	+
D‐Galactose	+	−	+	+
3‐Methyl glucose	−	+	w	−
L‐Rhamnose	−	+	−	−
D‐Sorbitol	+	−	+	+
D‐Aspartic acid	−	+	−	+
Glycyl‐L‐proline	−	+	+	−
L‐Arginine	−	+	−	+
L‐Aspartic acid	−	+	+	+
L‐Pyroglutamic acid	−	+	−	−
*p*‐Hydroxy‐phenylacetic acid	+	−	+	−
D‐Lactic acid methyl ester	−	+	−	−
L‐Lactic acid	+	+	−	+
Bromo‐succinic acid	+	−	+	−
Tween 40	−	+	−	−
*α*‐Hydroxy‐butyric acid	−	+	+	−
Sensitive to:				
Lincomycin	−	−	−	+
Guanidine HCl	+	+	−	+

Note

Strains: 1, strain D4M1^T^; 2, *Oceanicella actignis* DSM 22673^T^; 3, *Thioclava electrotropha* DSM 103712^T^; 4, *Oceanibium sediminis* MCCC 1H00233^T^. All data were experimentally determined in this study under the same conditions. Characteristics are scored as: +, positive; ‐, negative; w, weakly positive.

### 3.3 Fatty acids, polar lipids, and quinone composition

The predominant fatty acid of strain D4M1^T^ was summed feature 8 (41.7%, C_18:1_ *ω*7*c*/C_18:1_ *ω*6*c*) and C_16:0_ (36.9%) (Table A1). There were obvious differences in fatty acid profile between strain D4M1^T^ and reference strains DSM 22673^T^ and DSM 103712^T^. C_18:1_ *ω*7*c*/C_18:1_ *ω*6*c* were present in a much higher amount in reference strains DSM 22673^T^ and DSM 103712^T^ than in strain D4M1^T^, but the amount of C_16:0_ was much lower in the reference strains than in strain D4M1^T^.

**Figure A4:**
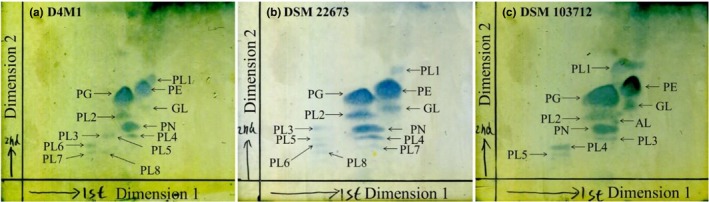
Polar lipids of strain D4M1^T^ (a), *Oceanicella actignis* LMG 25334^T^ (b) and *Thioclava electrotropha* DSM 103712^T^ (c) at the optimum growth conditions. AL, aminolipid; GL, glycolipid; PE, phosphatidylethanolamine; PG, phosphatidylglycerol; PL, phospholipid; PN, aminophospholipid

The major isoprenoid quinone of strain D4M1^T^ was ubiquinone 10 (Q‐10), which was the same as its related taxa in the family *Rhodobacteraceae* (Albuquerque et al., 2012; Y. Q. Chang, Meng, Du, & Du, 2019; Lai et al., 2014). The polar lipids of strain D4M1^T^ consisted of phosphatidylglycerol (PG), phosphatidylethanolamine (PE), an unidentified aminophospholipid (PN), an unidentified glycolipid (GL), and several unidentified phospholipids (PL) as shown in Figure A4, which were similar to those of reference strains DSM 22673^T^ and DSM 103712^T^, except some minor differences in unidentified phospholipids.

### 3.4 Genome annotation and analysis

The genome was shown to encode 4,942 predicted genes including 4,855 protein‐coding genes, 12 rRNAs (four 5S rRNA, four 16S rRNA, and four 23S rRNA), 57 tRNAs, and 9 ncRNAs. Complete genome analysis revealed that the 4,855 protein‐coding genes constituted 98.2% of the total genes in the genome, but only 79.3% were predicted with functions. Furthermore, there were 4,057 genes (82.1%) assigned to 24 different clusters of orthologous groups (COGs, Table A2), 1,489 genes (30.1%) connected to KEGG pathways, and 1,106 genes (22.4%) connected to MetaCyc pathways.

Analysis of the complete genome indicated the presence of different genes that are most likely linked to life at high pressure. Microbes are thought to preserve membrane fluidization and functionality at high pressure and low temperature in the deep sea by increasing the proportion of unsaturated fatty acids in their membrane lipids (Cao et al., 2015; Simonato et al., 2006). Strain D4M1^T^ contains high proportions of monounsaturated fatty acids, summed feature 8 (41.7%, C_18:1_ *ω*7*c*/C_18:1_ *ω*6*c*), probably for improving membrane piezo‐adaptation. Genomic analysis showed the presence of thirty‐seven genes involved in biosynthesis of unsaturated fatty acids, including four fatty acid desaturase genes (Table A3). Pressure‐induced chaperones proposed to help in maintaining protein folding (Oger & Jebbar, 2010) were also encoded adjacent to the unsaturated fatty acids biosynthesis genes in D4M1^T^ genome, including the OmpH which was thought to function as a nutrient transporter in nutrient‐limited deep sea (Table A3).

It is well known that many piezophiles change their respiratory chains in order to adapt to pressure (Oger & Jebbar, 2010). The genome was found to contain genes encoding cytochrome *bd*‐type quinol oxidase and cytochrome *cbb* protein complex (Table A3), which were involved in specific piezo‐adaptations in respiratory chain (Chikuma, Kasahara, Kato, & Tamegai, 2007; Qureshi, Kato, & Horikoshi, 1998). F_1_F_0_ ATP‐synthase was shown to facilitate energy‐yielding processes in high‐pressure adaptation (Souza, Creczynski‐Pasa, Scofano, Graber, & Mignaco, 2004). It was remarkable that two sets of the F_1_F_0_ ATP‐synthase genes were identified in the genome of strain D4M1^T^ (Table A3).

Deep‐sea bacteria were also found to accumulate protein‐stabilizing solutes at high pressure, such as piezolytes *β*‐hydroxybutyrate (*β*‐HB) and oligomers of *β*‐HB (Martin, Bartlett, & Roberts, 2002). PHB was detected in the cells of strain D4M1^T^ in this study, and genes that encoded the enzymes required for *β*‐HB and PHB synthesis were present in the genome, including 1 *β*‐HB dehydrogenase and 3 polyhydroxyalkanoate synthase genes (Table A3). The PHB inside the cells could also serve as intracellular carbon and energy reserves, which have been linked to pressure adaptation (Martin et al., 2002; Methe et al., 2005). Genes involved in biosynthesis and transport of compatible solutes, such as glycine betaine, were also identified in the genome, including genes encoding choline dehydrogenase and transcriptional repressor BetI (Table A3). It was suggested that trehalose protects proteins and cellular membranes from inactivation or denaturation caused by a variety of stress conditions, including high hydrostatic pressure (Simonato et al., 2006). Nineteen genes in the genome were predicted to encode trehalose biosynthesis and trehalose‐specific transporters (Table A3), which were probably involved in pressure adaptation.

Additionally, the genome of D4M1^T^ has six copies of *glnA*, including the counterpart of the pressure‐upregulated *glnA* (IMG Gene OID: 2828515862) in piezophile *Shewanella violacea* DSS12 (Ikegami, Nakasone, Kato, Nakamura, et al., 2000). Furthermore, the pressure‐regulated regulator *ntrBC* in *S*. *violacea* DSS12 was also identified in the genome of D4M1^T^ (Table A3), which was predicted to play a role in activation of transcription of pressure‐regulated promoters (Ikegami, Nakasone, Kato, Usami, & Horikoshi, 2000).

The increasing number of rRNA operons in a bacterial genome was previously proposed to represent a strategy for adapting to specific selective pressures from the environment (Klappenbach, Dunbar, & Schmidt, 2000). The genome of the strain was found to contain four rRNA operons (Table 1), which may correlate with the adaptation to the deep‐sea environment. Pressure is thermodynamically coupled to temperature. One “universal” response to environmental pressures is the biosynthesis of stress proteins (Kültz, 2005). The genome encoded 6 heat shock protein genes and 4 cold shock protein genes (Table A3), which were previously reported to be induced when exposed to high pressure (Simonato et al., 2006). Our results suggest that hydrostatic pressure is an important environmental stress that drives the adaptation of heat shock protein genes and cold shock protein genes in deep‐sea microorganisms.

The genome analysis revealed insights into the piezophilic lifestyle of the novel isolate and provided a reference for further phylogenomic, comparative genomic, and functional studies of the relative species in the deep ocean. However, further specific experiments need to be addressed in the future to find out the precise function of the genes involved in high‐pressure adaptation and the molecular adaptation mechanisms.

## 4 CONCLUSION

Strain D4M1^T^ exhibits the typical characteristics of the family *Rhodobacteraceae*, but it is also distinguishable from its closest relatives in the phylogenetic analysis of 16S rRNA gene sequence, the phylogenomic analysis based on whole‐genome protein sequences, the fatty acids profiles, the enzyme activities, the carbon utilization, the G + C contents, and the low 16S rRNA gene sequence similarity (≤95.8%) to the type species of the closely related genera of the family *Rhodobacteraceae*. Therefore, from the polyphasic evidence, strain D4M1^T^ represents a novel species of a novel genus for which the name *Paraoceanicella profunda* gen. nov., sp. nov. is proposed.

### 4.1 Description of *Paraoceanicella* gen. nov

*Paraoceanicella* (Pa.ra.o.ce.a.ni.cel'la. Gr. prep. para, beside, alongside of; N. L. fem. n. *Oceanicella*, a bacterial generic name; N. L. fem. n. *Paraoceanicella*, a genus adjacent to *Oceanicella*).

Cells are aerobic, Gram‐stain‐negative, oxidase‐ and catalase‐positive, short rods (1.0–1.5 × 0.6–0.8 μm). The G + C content of the genomic DNA of the type strain of the type species is 70.2 mol%. The predominant fatty acids are summed feature 8 (C_18:1_ *ω*7*c*/C_18:1_ *ω*6*c*), and C_16:0_. PG, PE, and an unidentified PN are the predominant polar lipids. Q‐10 is the major isoprenoid quinone.

The type species is *Paraoceanicella profunda*.

### 4.2 Description of *Paraoceanicella profunda* sp. nov

*Paraoceanicella profunda* (pro.fun'da. L. adj. *profunda* from the deep).

Cells are aerobic, Gram‐stain‐negative, oxidase‐ and catalase‐positive, short rods (1.0–1.5 × 0.6–0.8 μm). Growth occurs at salinities from 0.5% to 8.0% (optimum 3.0%), from pH 5.0 to 8.0 (optimum 6.5), and at temperatures between 10 and 45°C (optimum 37°C). Anaerobic growth does not occur in LB medium nor in LB medium supplemented with 10 mM of Na_2_SO_4_ or NaNO_3_. The optimum pressure for growth was 10 MPa with tolerance up to 70 MPa. Positive for nitrate reduction, alkaline phosphatase, esterase(C4), esterase lipase (C8), lipase (C14), leucine arylamidase, valine arylamidase, cystine arylamidase, *α*‐chymotrypsin, acid phosphatase, naphthol‐AS‐Bl‐phosphohydrolase, *α*‐glucosidase, arginine dihydrolase, gelatin hydrolysis and urease activities; negative for trypsin, *α*‐galactosidase, *β*‐galactosidase, *β*‐glucuronidase, *β*‐glucosidase, beta‐glucosidase (aesculin hydrolysis), *N*‐acetyl‐*β*‐glucosaminidase, *α*‐mannosidase, *α*‐fucosidase, indole production, or D‐glucose fermentation. Utilizes the following carbon sources: D‐glucose, L‐arabinose, D‐sorbitol, D‐mannitol, D‐arabitol, malic acid, potassium gluconate, D‐fructose, D‐fructose‐6‐PO_4_, D‐galactose, D‐fucose, L‐fucose, glycerol, L‐alanine, L‐glutamic acid, D‐galacturonic acid, L‐galactonic acid lactone, D‐gluconic acid, D‐glucuronic acid, glucuronamide, *p*‐hydroxy‐phenylacetic acid, methyl pyruvate, *α*‐keto‐glutaric acid, bromo‐succinic acid, *γ*‐amino‐butyric acid, *β*‐hydroxy‐D,L‐butyric acid, L‐serine, glucuronamide, quinic acid, D‐saccharic acid, L‐lactic acid, acetoacetic acid, propionic acid, and acetic acid. The predominant fatty acid is summed feature 8 (C_18:1_ *ω*7*c*/C_18:1_ *ω*6*c*) and C_16:0_. Q‐10 is the major isoprenoid quinone. The predominant polar lipids consist of PG, PE, and an unidentified PN. The G + C content of the genomic DNA is 70.2 mol%.

The type strain D4M1^T^ (=MCCC 1K03820^T^ = KCTC 72285^T^) was cultured from a deep‐water sample obtained at a depth of 10,890 m of the Mariana Trench (142.4°E, 11.4°N; site MT). The 16S rRNA and genome sequences are submitted to GenBank under accession numbers MK909903 and CP040818–CP040824, respectively.

## ETHICS STATEMENT

None required.

## CONFLICT OF INTERESTS

None declared.

## AUTHOR CONTRIBUTIONS

JC supervised the project. PL, WD, and QL carried out the experiments. LP and JC analyzed the data. PL, JC, and JF wrote the manuscript with support from RL, YW, LW, and ZX.

## Data Availability

The GenBank [/EMBL/DDBJ] accession numbers for the 16S rRNA gene sequence of strain D4M1^T^ are MK909903: https://www.ncbi.nlm.nih.gov/nuccore/MK909903. The assembled and annotated genome of D4M1^T^ described in this paper has been deposited in GenBank (accession number: CP040818‐CP040824: https://www.ncbi.nlm.nih.gov/assembly/GCA_005887635.2) and JGI portal (GOLD ID: Gp0432545, https://gold.jgi.doe.gov/project?xml:id=Gp0432545; IMG Taxon ID: 2828513066, https://img.jgi.doe.gov/cgi-bin/m/main.cgi?section=TaxonDetail&page=taxonDetail&taxon_oxml:id=2828513066).

## APPENDIX 1

**Table A1:** Fatty acid contents of strain D4M1^T^ and closely related species

Fatty acids	1	2	3
C_10:0_ 3‐OH	—	—	1.9
iso‐C_11:0_ 3‐OH	1.6	—	—
iso‐C_12:0_	1.3	0.2	0.2
C_12:0_	—	—	0.2
Summed feature 2	3.7	2.5	—
Summed feature 3	1.7	0.2	2.0
C_16:0_	36.9	4.0	2.4
C_17:1_ *ω*7*c*	0.8	—	—
C_17:1_ *ω*8*c*	—	1.0	0.2
C_17:0_	—	2.5	1.4
Summed feature 8	41.7	65.1	82.7
C_18:0_	7.5	10.0	6.0
C_18:1_ *ω*7*c* 11‐methyl	3.5	10.2	0.6
C_18:0_ 3‐OH	—	2.9	0.4

Note

Taxa: 1, strain D4M1^T^; 2, *Oceanicella actignis* LMG 25334^T^; 3, *Thioclava electrotropha* DSM 103712^T^; data of all strains were from this study under the same condition. Values are percentages of total fatty acids; —, not detected. *Summed features represent groups of two or three fatty acids which could not be separated by GLC with the MIDI system. Summed feature 2, C_14:0_ 3‐OH/iso‐C_16:1_ I; summed feature 3, C_16:1_ *ω*7*c*/*ω*6*c*; summed feature 8, C_18:1_ *ω*7*c*/*ω*6*c*

**Table A2:** COG categories of the predicted W43^T^ genes

COG categories	Code	Gene count	Percent (%)
Amino acid transport and metabolism	E	646	13.36
Carbohydrate transport and metabolism	G	334	6.91
Cell cycle control, cell division, chromosome partitioning	D	44	0.91
Cell motility	N	65	1.34
Cell wall/membrane/envelope biogenesis	M	219	4.53
Chromatin structure and dynamics	B	5	0.10
Coenzyme transport and metabolism	H	213	4.40
Cytoskeleton	Z	1	0.02
Defense mechanisms	V	93	1.92
Energy production and conversion	C	279	5.77
Extracellular structures	W	5	0.10
Function unknown	S	261	5.40
General function prediction only	R	542	11.21
Inorganic ion transport and metabolism	P	306	6.33
Intracellular trafficking, secretion, and vesicular transport	U	58	1.20
Lipid transport and metabolism	I	254	5.25
Mobilome: prophages, transposons	X	52	1.08
Nucleotide transport and metabolism	F	106	2.19
Posttranslational modification, protein turnover, chaperones	O	175	3.62
Replication, recombination, and repair	L	134	2.77
Secondary metabolites biosynthesis, transport, and catabolism	Q	225	4.65
Signal transduction mechanisms	T	193	3.99
Transcription	K	407	8.42
Translation, ribosomal structure, and biogenesis	J	219	4.53

**Table A3:** Genes in the D4M1^T^ genome involved in high‐pressure adaptation

IMG gene OID	Locus tag	Protein
Biosynthesis of unsaturated fatty acids		
2828513318	Ga0392526_253	3‐oxoacyl‐[acyl‐carrier protein] reductase
2828513436	Ga0392526_371	Glycerol‐3‐phosphate acyltransferase PlsY
2828513500	Ga0392526_436	1‐acyl‐sn‐glycerol‐3‐phosphate acyltransferase
2828513541	Ga0392526_477	acyl‐CoA thioesterase‐1
2828513884	Ga0392526_820	3‐oxoacyl‐[acyl‐carrier protein] reductase
2828514031	Ga0392526_967	3‐oxoacyl‐[acyl‐carrier protein] reductase
2828514243	Ga0392526_1179	3‐oxoacyl‐[acyl‐carrier protein] reductase
2828514304	Ga0392526_1240	fatty acid desaturase
2828514475	Ga0392526_1411	acyl transferase domain‐containing protein/NADPH:quinone reductase‐like Zn‐dependent oxidoreductase/acyl carrier protein
2828514527	Ga0392526_1463	enoyl‐[acyl‐carrier protein] reductase I
2828514528	Ga0392526_1464	3‐oxoacyl‐[acyl‐carrier‐protein] synthase‐1
2828514529	Ga0392526_1465	3‐hydroxyacyl‐[acyl‐carrier protein] dehydratase/trans‐2‐decenoyl‐[acyl‐carrier protein] isomerase
2828514544	Ga0392526_1480	acyl‐CoA thioesterase YciA
2828514651	Ga0392526_1587	fatty acid desaturase
2828514809	Ga0392526_1745	fatty acid desaturase
2828515056	Ga0392526_1992	enoyl‐CoA hydratase
2828515178	Ga0392526_2114	1‐acyl‐sn‐glycerol‐3‐phosphate acyltransferase
2828515283	Ga0392526_2219	3‐hydroxyacyl‐CoA dehydrogenase/enoyl‐CoA hydratase/3‐hydroxybutyryl‐CoA epimerase
2828515303	Ga0392526_2239	1‐acyl‐sn‐glycerol‐3‐phosphate acyltransferase
2828515340	Ga0392526_2276	long‐chain acyl‐CoA synthetase
2828515835	Ga0392526_2771	3‐hydroxyacyl‐[acyl‐carrier‐protein] dehydratase
2828515979	Ga0392526_2915	3‐oxoacyl‐[acyl‐carrier protein] reductase
2828516231	Ga0392526_3167	3‐oxoacyl‐[acyl‐carrier‐protein] synthase II
2828516233	Ga0392526_3169	3‐oxoacyl‐[acyl‐carrier protein] reductase
2828516304	Ga0392526_3240	3‐oxoacyl‐[acyl‐carrier protein] reductase
2828516395	Ga0392526_3331	3‐oxoacyl‐[acyl‐carrier protein] reductase
2828516464	Ga0392526_3400	3‐oxoacyl‐[acyl‐carrier protein] reductase
2828516468	Ga0392526_3404	3‐oxoacyl‐[acyl‐carrier protein] reductase
2828516565	Ga0392526_3501	Glycerol‐3‐phosphate acyltransferase PlsX
2828516609	Ga0392526_3545	enoyl‐[acyl‐carrier protein] reductase I
2828516730	Ga0392526_3666	enoyl‐CoA hydratase
2828516791	Ga0392526_3727	3‐oxoacyl‐[acyl‐carrier protein] reductase
2828516807	Ga0392526_3743	3‐oxoacyl‐[acyl‐carrier protein] reductase
2828516926	Ga0392526_3862	enoyl‐CoA hydratase
2828517203	Ga0392526_4139	enoyl‐[acyl‐carrier protein] reductase I
2828517211	Ga0392526_4147	fatty acid desaturase
2828517574	Ga0392526_4510	3‐oxoacyl‐[acyl‐carrier protein] reductase
Chaperone		
2828513714	Ga0392526_650	molecular chaperone DnaK
2828513716	Ga0392526_652	molecular chaperone DnaJ
2828515281	Ga0392526_2217	Zn‐dependent protease with chaperone function
2828515832	Ga0392526_2768	regulator of sigma E protease
2828515833	Ga0392526_2769	outer membrane protein insertion porin family
2828515834	Ga0392526_2770	Skp family chaperone for outer membrane proteins, OmpH
2828516688	Ga0392526_3624	HSP20 family molecular chaperone IbpA
2828516690	Ga0392526_3626	molecular chaperone Hsp33
Respiratory chain		
2828513683	Ga0392526_619	cytochrome d ubiquinol oxidase subunit I
2828513684	Ga0392526_620	cytochrome d ubiquinol oxidase subunit II
2828515783	Ga0392526_2719	cbb3‐type cytochrome oxidase maturation protein
2828515787	Ga0392526_2723	cytochrome c oxidase accessory protein FixG
2828515788	Ga0392526_2724	uncharacterized membrane protein
2828515789	Ga0392526_2725	cytochrome c oxidase cbb3‐type subunit 3
2828515790	Ga0392526_2726	cytochrome c oxidase cbb3‐type subunit 4
2828515791	Ga0392526_2727	cytochrome c oxidase cbb3‐type subunit 2
2828515792	Ga0392526_2728	cytochrome c oxidase cbb3‐type subunit 1
F1F0 ATP‐synthase		
2828514969	Ga0392526_1905	F1F0 ATP‐synthase subunit delta
2828514970	Ga0392526_1906	F1F0 ATP‐synthase subunit alpha
2828514971	Ga0392526_1907	F1F0 ATP‐synthase subunit gamma
2828514972	Ga0392526_1908	F1F0 ATP‐synthase subunit beta
2828514973	Ga0392526_1909	F1F0 ATP‐synthase subunit epsilon
2828515088	Ga0392526_2024	F1F0 ATP‐synthase, membrane subunit b
2828515089	Ga0392526_2025	F1F0 ATP‐synthase, membrane subunit b
2828515090	Ga0392526_2026	F1F0 ATP‐synthase, membrane subunit c
2828515091	Ga0392526_2027	F1F0 ATP‐synthase, membrane subunit a
2828515092	Ga0392526_2028	F1F0 ATP‐synthaseprotein I
2828516954	Ga0392526_3890	F1F0 ATP‐synthase subunit beta
2828516955	Ga0392526_3891	F1F0 ATP‐synthase subunit epsilon
2828516956	Ga0392526_3892	F1F0 ATP‐synthaseprotein I
2828516957	Ga0392526_3893	F1F0 ATP‐synthase subunit 2
2828516958	Ga0392526_3894	F1F0 ATP‐synthase subunit a
2828516959	Ga0392526_3895	F1F0 ATP‐synthase subunit c
2828516960	Ga0392526_3896	F1F0 ATP‐synthase subunit b
2828516961	Ga0392526_3897	F1F0 ATP‐synthase subunit alpha
2828516962	Ga0392526_3898	F1F0 ATP‐synthase subunit gamma
PHA/PHB synthesis		
2828513971	Ga0392526_907	3‐hydroxybutyrate dehydrogenase
2828514023	Ga0392526_959	putative acetyltransferase
2828514219	Ga0392526_1155	hydroxymethylglutaryl‐CoA lyase
2828514509	Ga0392526_1445	apolipoprotein N‐acyltransferase
2828514635	Ga0392526_1571	polyhydroxyalkanoate synthase
2828514700	Ga0392526_1636	polyhydroxybutyrate depolymerase
2828514800	Ga0392526_1736	poly(3‐hydroxybutyrate) depolymerase
2828514849	Ga0392526_1785	polyhydroxyalkanoate depolymerase
2828514923	Ga0392526_1859	3‐hydroxyacyl‐CoA dehydrogenase
2828515161	Ga0392526_2097	acetoacetyl‐CoA reductase
2828515162	Ga0392526_2098	acetyl‐CoA C‐acetyltransferase
2828515163	Ga0392526_2099	polyhydroxyalkanoate synthase
2828515164	Ga0392526_2100	polyhydroxyalkanoate synthesis repressor PhaR
2828515217	Ga0392526_2153	3‐hydroxybutyryl‐CoA dehydrogenase
2828515283	Ga0392526_2219	3‐hydroxyacyl‐CoA dehydrogenase/enoyl‐CoA hydratase/3‐hydroxybutyryl‐CoA epimerase
2828515284	Ga0392526_2220	acetyl‐CoA C‐acetyltransferase
2828515305	Ga0392526_2241	3‐hydroxybutyryl‐CoA dehydratase
2828515724	Ga0392526_2660	3‐hydroxybutyryl‐CoA dehydrogenase
2828516037	Ga0392526_2973	3‐oxoacyl‐(acyl‐carrier‐protein) synthase/nodulation protein E
2828516295	Ga0392526_3231	hydroxymethylglutaryl‐CoA lyase
2828516711	Ga0392526_3647	putative acetyltransferase
2828516927	Ga0392526_3863	3‐hydroxybutyryl‐CoA dehydrogenase
2828517202	Ga0392526_4138	polyhydroxyalkanoate synthase
2828517335	Ga0392526_4271	acetyl‐CoA C‐acetyltransferase
2828517488	Ga0392526_4424	hydroxymethylglutaryl‐CoA lyase
2828517899	Ga0392526_4835	apolipoprotein N‐acyltransferase
2828517938	Ga0392526_4874	acetyl‐CoA C‐acetyltransferase
Glutamine synthesis and regulation		
2828513735	Ga0392526_671	glutamine synthetase
2828514055	Ga0392526_991	glutamine synthetase
2828514780	Ga0392526_1716	glutamine synthetase
2828514889	Ga0392526_1825	glutamine synthetase
2828515862	Ga0392526_2798	glutamine synthetase
2828516007	Ga0392526_2943	ntrX two‐component system, NtrC family, nitrogen regulation response regulator NtrX
2828516008	Ga0392526_2944	ntrY two‐component system, NtrC family, nitrogen regulation sensor histidine kinase NtrY
2828516009	Ga0392526_2945	ntrC two‐component system, NtrC family, nitrogen regulation response regulator GlnG
2828516010	Ga0392526_2946	ntrB two‐component system, NtrC family, nitrogen regulation sensor histidine kinase GlnL
2828516194	Ga0392526_3130	glutamine synthetase
Betaine and trehalose biosynthesis and transport		
2828513193	Ga0392526_128	choline dehydrogenase
2828513583	Ga0392526_519	choline dehydrogenase
2828513584	Ga0392526_520	choline‐sulfatase
2828513585	Ga0392526_521	TetR/AcrR family transcriptional repressor of bet genes
2828513586	Ga0392526_522	glycine betaine/proline transport system substrate‐binding protein
2828513587	Ga0392526_523	glycine betaine/proline transport system permease protein
2828513588	Ga0392526_524	glycine betaine/proline transport system ATP‐binding protein
2828513835	Ga0392526_771	glycine betaine/proline transport system substrate‐binding protein
2828513836	Ga0392526_772	drug/metabolite transporter (DMT)‐like permease
2828513837	Ga0392526_773	DNA‐binding Lrp family transcriptional regulator
2828513838	Ga0392526_774	DNA‐binding HxlR family transcriptional regulator
2828514400	Ga0392526_1336	choline monooxygenase
2828515533	Ga0392526_2469	BCCT family betaine/carnitine transporter
2828516142	Ga0392526_3078	glycine betaine/proline transport system substrate‐binding protein
2828516143	Ga0392526_3079	glycine betaine/proline transport system permease protein
2828516144	Ga0392526_3080	glycine betaine/proline transport system ATP‐binding protein
2828516539	Ga0392526_3475	choline dehydrogenase
2828514833	Ga0392526_1769	Acetyltransferase (isoleucine patch superfamily)
2828515886	Ga0392526_2822	glucosylglycerol‐phosphate synthase
2828516592	Ga0392526_3528	trehalose/maltose transport system substrate‐binding protein
2828516593	Ga0392526_3529	trehalose/maltose transport system permease protein
2828516594	Ga0392526_3530	trehalose/maltose transport system permease protein
2828516595	Ga0392526_3531	ABC‐type sugar transport system ATPase subunit
2828517438	Ga0392526_4374	trehalose 6‐phosphate synthase
2828517439	Ga0392526_4375	trehalose 6‐phosphate phosphatase
2828517440	Ga0392526_4376	(1‐>4)‐alpha‐D‐glucan 1‐alpha‐D‐glucosylmutase
2828517441	Ga0392526_4377	4‐alpha‐glucanotransferase
2828517442	Ga0392526_4378	maltooligosyltrehalose trehalohydrolase
2828517443	Ga0392526_4379	glycogen operon protein
2828517444	Ga0392526_4380	1,4‐alpha‐glucan branching enzyme
2828517445	Ga0392526_4381	maltose alpha‐D‐glucosyltransferase/alpha‐amylase
2828517644	Ga0392526_4580	multiple sugar transport system ATP‐binding protein
2828517645	Ga0392526_4581	multiple sugar transport system permease protein
2828517646	Ga0392526_4582	multiple sugar transport system permease protein
2828517647	Ga0392526_4583	multiple sugar transport system substrate‐binding protein
2828517648	Ga0392526_4584	LacI family transcriptional regulator
Cold and heat shock proteins		
2828513475	Ga0392526_411	CspA family cold shock protein
2828513528	Ga0392526_464	ribosome‐associated heat shock protein Hsp15
2828513634	Ga0392526_570	heat shock protein HspQ
2828514209	Ga0392526_1145	heat shock gene repressor HrcA
2828514210	Ga0392526_1146	molecular chaperone GrpE (heat shock protein)
2828514746	Ga0392526_1682	heat shock protein HtpX
2828514758	Ga0392526_1694	CspA family cold shock protein
2828515042	Ga0392526_1978	CspA family cold shock protein
2828515213	Ga0392526_2149	heat shock protein HslJ
2828516605	Ga0392526_3541	cold shock CspA family protein
